# Role of liposome and peptide in the synergistic enhancement of transfection with a lipopolyplex vector

**DOI:** 10.1038/srep09292

**Published:** 2015-03-19

**Authors:** Mustafa M. Munye, Jascindra Ravi, Aristides D. Tagalakis, David McCarthy, Maxim G. Ryadnov, Stephen L. Hart

**Affiliations:** 1 UCL Institute of Child Health, 30 Guilford Street, London, WC1N 1EH, United Kingdom; 2 National Physical Laboratory, Teddington, Middlesex, TW11 0LW, United Kingdom; 3 UCL School of Pharmacy, 29–39 Brunswick Square, London, WC1N 1AX, United Kingdom

## Abstract

Lipopolyplexes are of widespread interest for gene therapy due to their multifunctionality and high transfection efficiencies. Here we compared the biological and biophysical properties of a lipopolyplex formulation with its lipoplex and polyplex equivalents to assess the role of the lipid and peptide components in the formation and function of the lipopolyplex formulation. We show that peptide efficiently packaged plasmid DNA forming spherical, highly cationic nanocomplexes that are taken up efficiently by cells. However, transgene expression was poor, most likely due to endosomal degradation since the polyplex lacks membrane trafficking properties. In addition the strong peptide-DNA interaction may prevent plasmid release from the complex and so limit plasmid DNA availability. Lipid/DNA lipoplexes, on the other hand, produced aggregated masses that showed poorer cellular uptake than the polyplex but contrastingly greater levels of transgene expression. This may be due to the greater ability of lipoplexes relative to polyplexes to promote endosomal escape. Lipopolyplex formulations formed spherical, cationic nanocomplexes with efficient cellular uptake and significantly enhanced transfection efficiency. The lipopolyplexes combined the optimal features of lipoplexes and polyplexes showing optimal cell uptake, endosomal escape and availability of plasmid for transcription, thus explaining the synergistic increase in transfection efficiency.

Gene therapy involves the use of genes for therapeutic purposes and is being evaluated for a number of diseases. Gene delivery requires a suitable carrier system for which viral vectors are generally more efficient than their non-viral nanoparticle counterparts. However, the latter are easier to prepare in large quantities, have a packaging capacity for a wider range of sizes of nucleic acid molecules, ranging from 20 nucleotides to tens of kilobases, and perhaps most importantly, are less immunogenic and so may be more useful for clinical applications that require repeated gene delivery^1^. However, whilst significant progress has been made in developing nanoparticles more efficient, multifunctional formulations are still required to accelerate their clinical progression for gene therapy.

Felgner et al. in 1987^2^ first reported that cationic liposomes could be formulated with plasmid DNA into self-assembling complexes that transfected cells while, in the same year, Wu and Wu showed that the cationic polymer poly-L-lysine formed transfection complexes when added to DNA^3^. A variety of lipoplexes and polyplexes have since been developed and optimized for gene transfer^4^,^5^. Lipopolyplex formulations that combine both liposomes and polymers have gained popularity in recent years as the mixture of components enables the formation of nanocomplexes with a wider range of functionalities than either lipoplexes or polyplexes, with higher transfection efficiencies^6^,^7^,^8^,^9^,^10^.

We have previously described LPD nanocomplex formulations for gene delivery comprising a mixture of cationic liposome (L; e.g., DOTMA/DOPE) and cationic targeting peptide (P) which self-assemble on mixing with DNA (D) at appropriate ratios^11^. Derivatives of LPD nanocomplexes have been used for *in vivo* gene delivery to lung^12^,^13^, vascular tissues^14^,^15^,^16^ and tumours^17^,^18^ achieving efficient gene transfer and displaying therapeutic effects in tumours^18^ and vasculature^15^,^16^. Importantly, LPD formulations were far more effective transfection agents than either PD or LD suggesting a synergistic interaction of components.

In the present study the aim was to investigate the basis of the synergistic increase in transfection efficiency of the LPD formulations compared to its liposome-DNA (LD) and peptide-DNA (PD) counterparts. As a model lipopolyplex we used an LPD formulation previously optimised for airway gene transfer^12^. The LPD vector was formulated with a liposome (L), made up of DHDTMA (1-Propanaminium, N,N,N-trimethyl-2,3-bis (11Z-hexadecenyloxy)-iodide) and DOPE lipids, a dual functioning DNA-condensing and receptor-targeted peptide (P), with the sequence K_16_GACSERSMNFCG^19^ (K_16_E) and DNA (D) formulated at an L:P:D weight ratio of 0.75:4:1. We performed detailed structural and functional analysis of the LPD formulation together with its LD and PD counterparts. We sought to interrogate the roles of the liposome and peptide in transfection in order to explain the synergy we and others have observed in transfections with lipopolyplexes. We aim to use this information to develop more improved nanoparticle formulations.

## Results

### In vitro transfections

**Figure 1:**
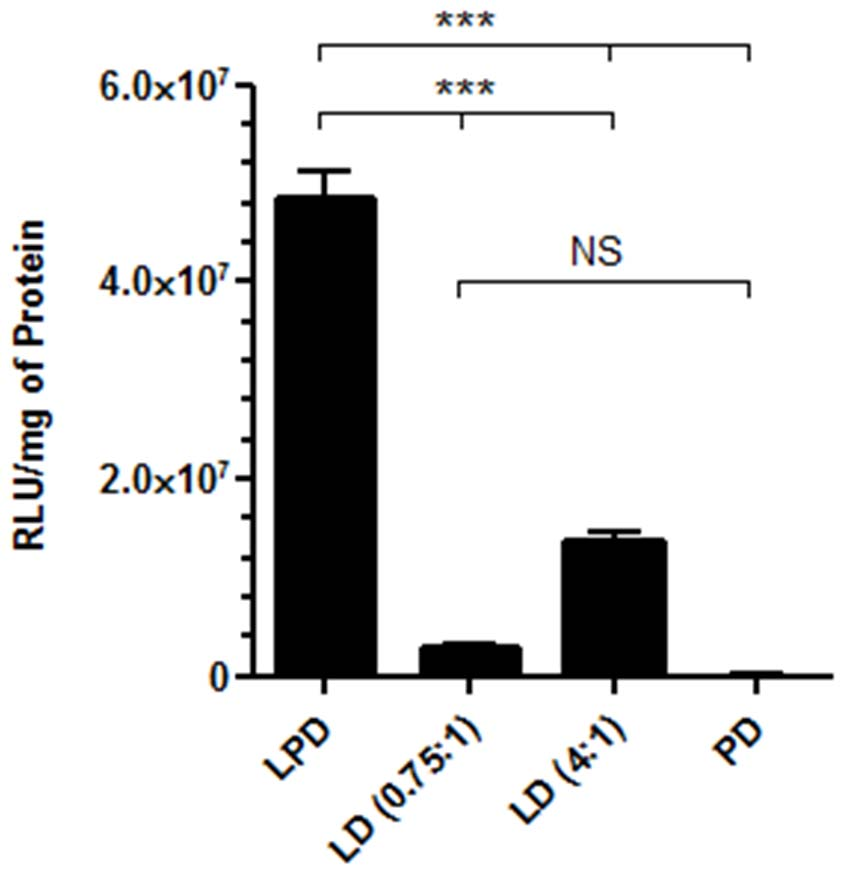
Luciferase activity, measured in relative light units (RLU) per mg of protein following transfection of 16HBE14o^-^ cells with pCI-Luc complexed into LPD, LD (0.75:1), LD (4:1) and PD nanocomplexes. Values are mean ± SEM; n = 5; NS, not significant, ***P < 0.001; one-way ANOVA with Bonferroni post-test analysis used to assess significance.

16HBE14o- cells were transfected with LPD, LD and PD formulations with pCI-Luc and luciferase expression assessed to compare their transfection efficiencies. Transfected luciferase activity using the LPD formulation was 3.5-fold higher than that achieved with the LD (4:1) formulation (Figure 1; p < 0.001), 15.9-fold greater than the LD (0.75:1) formulation (Figure 1; p < 0.001) and 1030.5-fold higher than with the PD formulation (Figure 1; p < 0.001). Moreover, LD (4:1) formulations, but not LD (0.75:1) formulations, gave significantly higher levels of luciferase activity than PD formulations (Figure 1; p < 0.001). Therefore, the ternary combination of liposome, peptide and DNA of LPD formulations displayed a synergistic increase in luciferase transgene expression compared to binary PD and LD formulations (Figure 1).

This synergy in transfection was also observed following HEK-293T and HT-1080 cell line transfections (Figure S1a and S1b, respectively). In both cases, transfected luciferase activity using the LPD formulation was around 3-fold higher than that achieved with the LD (4:1) formulation (Figure S1a and S1b; p < 0.001). LD (4:1) formulations, but not LD (0.75:1) formulations, again gave significantly higher levels of luciferase activity than PD formulations (Figure S1a and S1b; p < 0.001).

### Biological barriers to gene transfer

**Figure 2:**
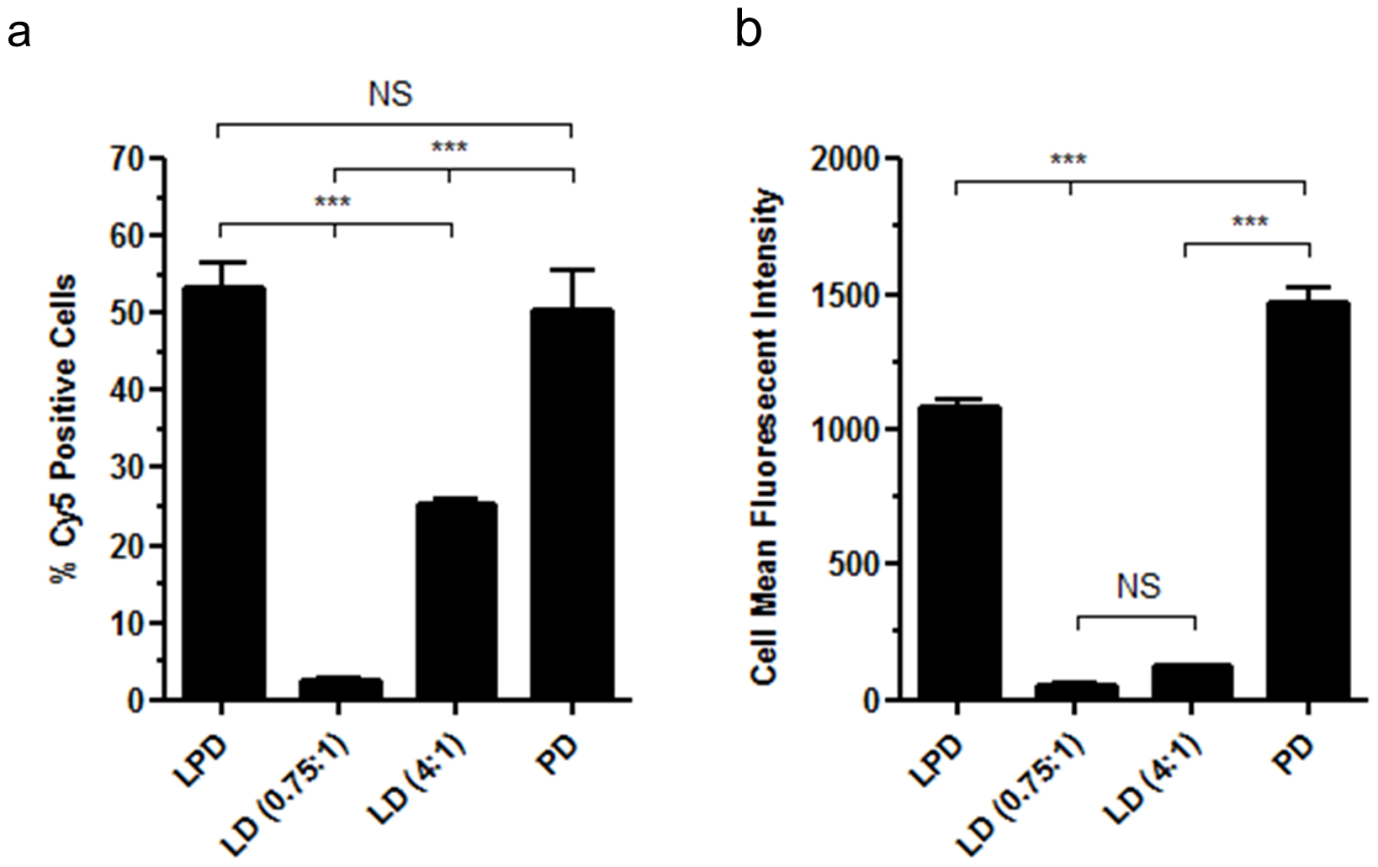
Flow cytometry analysis showing (a) the percentage of Cy5 positive cells and (b) the mean fluorescent intensity of Cy5 positive cells following a 6 hour incubation of 16HBE14o^-^ cells with Cy5 labelled pCI-Luc complexed into LD (0.75:1), LD (4:1), PD and LPD complexes. Values are mean ± SEM; n = 6; NS, not significant; ***P < 0.001; one-way ANOVA with Bonferroni post-test analysis used to assess significance.

The initial barriers to transfection are cell binding and uptake and differences in efficiencies of these processes could potentially explain differences in transfection efficiency of different nanocomplexes. To assess differences in vector uptake, 16HBE14o- cells were transfected with LPD, PD, and LD complexes formulated with Cy5-labelled pCI-Luc and plasmid uptake assessed by flow cytometry. With LD (0.75:1) complexes 2.5 ± 0.1% of cells showed uptake of plasmid DNA compared to 25.2 ± 0.9% with LD (4:1) complexes (Figure 2a; p < 0.001). In both cases LD complexes showed significantly lower levels of plasmid uptake than PD and LPD complexes, which showed uptake in 50.5 ± 5.0%, and 53.4 ± 3.1% of cells, respectively (Figure 2a; p < 0.001 for LD vs. LPD/PD), while the small difference in cell uptake between PD and LPD complexes was not significant. We also assessed the mean fluorescence intensity (MFI) of Cy5 labelled plasmid within the cells positive for Cy5 (Figure 2b). There was no significant difference in MFI when comparing the two LD complexes (MFI of 56.7 ± 4.8 and 126.8 ± 3.1 for LD (0.75:1) and LD (4:1), respectively) but both LD complexes showed a significantly lower MFI than LPD (MFI of 1080 ± 33.2) and PD complexes (MFI of 1472 ± 54.2 (Figure 2b; p < 0.001 for LD vs. LPD/PD). Whilst there was no significant difference between LPD and PD complexes in regards to percentage of transfected cells (Figure 2a), the latter did show significantly higher levels of Cy5 labelled plasmid within each transfected cell (Figure 2b; p < 0.001). Confocal microscopy images of transfected cells corroborated these findings and showed that uptake of Cy5 labelled plasmid was greatest in cells transfected with PD and LPD formulations and lowest with the LD (0.75:1) formulation (Figure S2).

Downstream of cell uptake vectors must escape the endosome compartment to avoid subsequent degradation in lysosomes. The DOPE lipid within the DHTDMA:DOPE liposome is known to aid in this endosomal escape and was crucial in producing efficient transgene expression with LPD and LD complexes as its replacement with the non-fusogenic DOPC lipid dramatically decreased transgene expression to PD transfection levels (Figure S3). It was clear then that vector uptake was the limiting step for LD transfections and endosomal escape the limiting step for PD transfections.

Having established the limiting steps to transfection with LD and PD complexes we next investigated whether differences in their biophysical properties could explain their different behaviour in cells.

### Morphology, size, and charge characterisation

**Figure 3:**
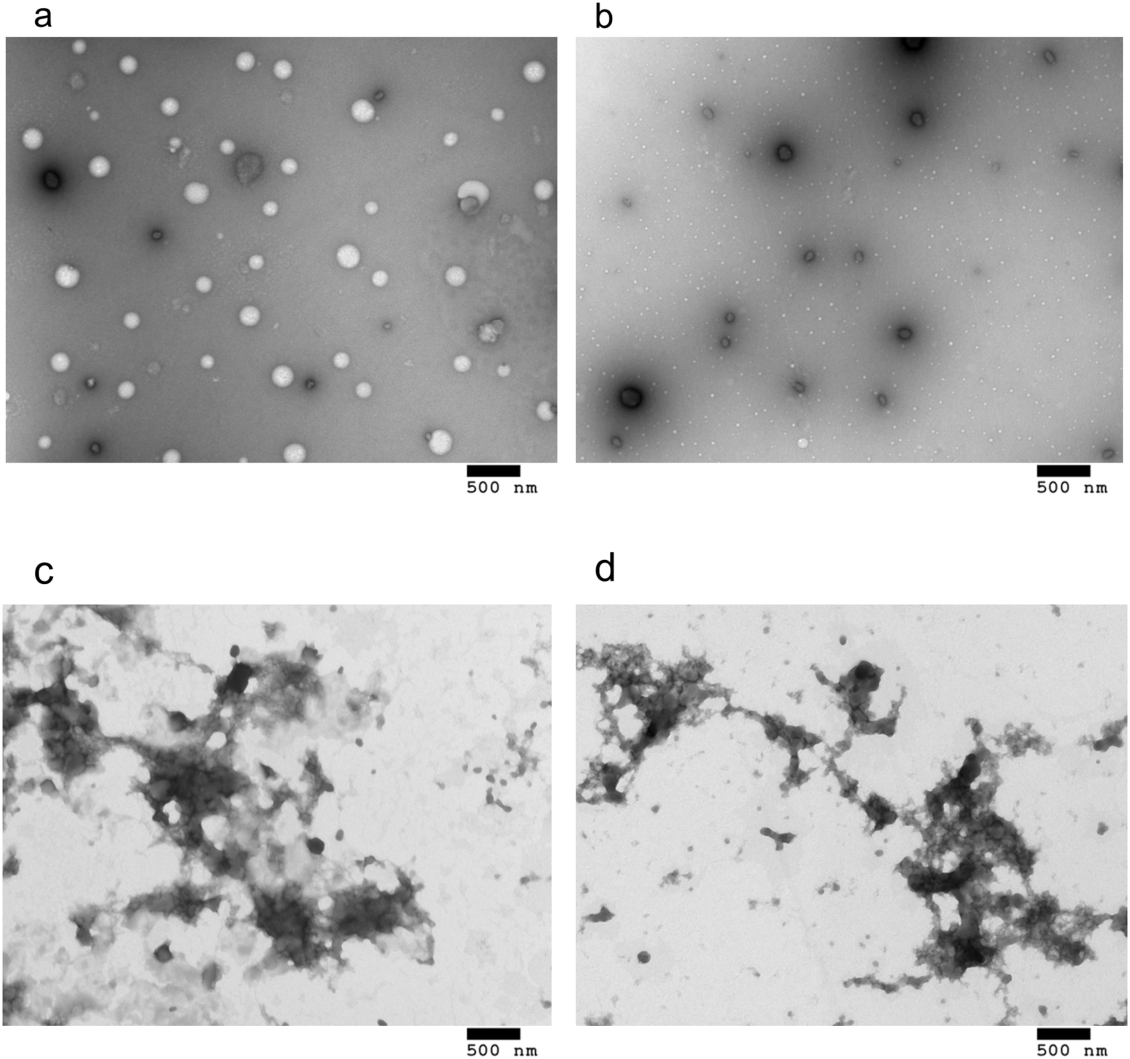
Transmission electron micrographs of (a) LPD, (b) PD, (c) LD (0.75:1) and (d) LD (4:1) complexes. Scale bars are 500 nm.

Particle morphology, size and surface charge are all important in determining cell uptake pathway and efficiency. Transmission electron microscopy (TEM) and dynamic light scattering (DLS) were used to investigate the morphology, size and charge of the different complexes. TEM showed that LPD complexes formed a heterogeneous population of roughly spherical particles with sizes around 100–200 nm in diameter (Figure 3a and S4a) while PD complexes were also roughly spherical though particle sizes were smaller than LPD with the majority falling within the range of 30–60 nm and a few larger particles with sizes of around 100 nm (Figure 3b and S4b). In contrast to LPD and PD complexes, LD mixtures at both the 0.75:1 and 4:1 liposome: DNA ratios appeared as aggregated multi-lamellar structures (Figure 3c and d; Figure S4c and d) with more rounded particles apparent at the 4:1 L:D ratio (Figure 3d).

DLS measurements of the four described complexes were consistent with the TEM measurements showing that the PD complexes were smallest followed by LPD, LD (0.75:1) and LD (4:1) complexes (Table 1). Zeta potential measurements for LPD, LD (4:1) and PD complexes were all cationic but LD (0.75:1) complexes showed a negative zeta potential suggesting that the DNA was not neutralized or condensed at all (Table 1). The liposome used in this study had a smaller hydrodynamic size than both LD (4:1) and LD (0.75:1) complexes as well as a higher surface charge. A mixture of liposome and peptide (LP) showed a similar hydrodynamic size to liposome alone (Table 1) suggesting no interaction between liposome and peptide.

### DNA condensation and packaging

Differences observed in the morphology, size and charge of the LD, PD and LPD formulations indicates differences in DNA condensation and packaging. DNA condensation was assessed using gel retardation and fluorescence quenching assays while DNA release from each nanocomplex was assessed by incubation with the highly anionic heparan sulphate to promote dissociation.

**Figure 4:**
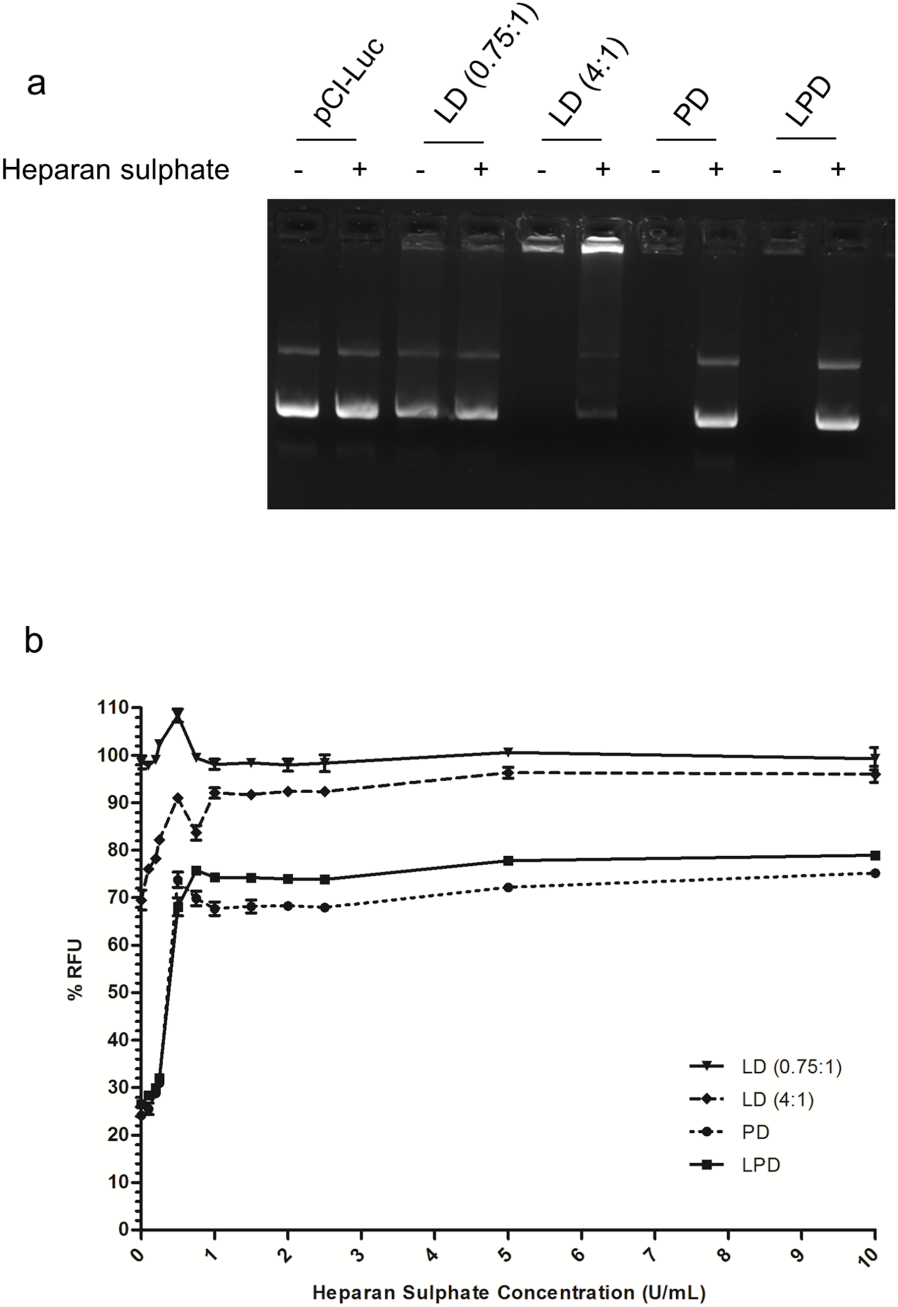
(a) Gel retardation assay showing 250 ng naked pCI-Luc, LD (0.75:1), LD (4:1), PD, and LPD complexes incubated for 1 hour at room temperature in the presence or absence of 20 U/mL heparan sulphate. (b) PicoGreen fluorescence quenching was used to assess the stability of the different complexes to heparan sulphate challenge as well as assess the quenching capabilities of the different complexes in the absence of heparan sulphate. The level of PicoGreen fluorescence was measured in relative fluorescence units (RFU) with naked PicoGreen labelled pCI-Luc plasmid set as 100% RFU. Values are mean ± SEM; n = 6.

With LD (0.75:1) complexes a significant amount of plasmid was not retarded indicating poor plasmid condensation while LD (4:1) complexes completely retarded plasmid DNA in the wells (Figure 4a). LPD and PD complexes also showed retarded plasmid mobility but with greater ethidium bromide exclusion than the LD (4:1) complexes as suggested by the fainter signal in the wells compared to the LD (4:1) complexes (Figure 4a). The observed differences in plasmid DNA condensation were further supported by quenching experiments using the PicoGreen assay (Figure 4b). When normalised to free plasmid 30.5 ± 1.2% of PicoGreen labelled plasmid was quenched in LD (4:1) complexes which contrasted with the 73.5 ± 0.1% and 75.9 ± 0.04% quenching of fluorescence seen when using LPD and PD complexes respectively. LD (0.75:1) complexes did not show any detectable quenching of PicoGreen labelled plasmid. Taken together the data indicated that LPD and PD complexes packaged plasmid DNA more efficiently than LD complexes.

Agarose gel analysis showed that heparan sulphate treatment liberated plasmid DNA from LPD and PD samples readily but less well from LD (4:1) complexes. The organisation and packaging of plasmid DNA is thus similar in LPD and PD complexes and dissimilar in LPD and LD (4:1) complexes. PicoGreen release studies showed that LPD and PD complexes were more stable than LD complexes in response to increasing concentrations of heparan sulphate. This study also revealed that PD complexes were more stable than LPD as less DNA was released from PDs by heparan sulphate incubation. Initial differences in levels of PicoGreen quenching of plasmid DNA were only 2.4% (Figure 4b) but this increased to 5.7% in 5 U/mL heparan sulphate (22.2% of fluorescence quenched for LPD and 27.9% for PD, p < 0.001; Figure 4b). Release of plasmid DNA may therefore be more efficient with the LPD complexes than with the PD complexes.

### Liposome, peptide, DNA interactions in forming LPD particles

Having established differences in the biophysical properties of LPD, LD and PD complexes we sought to investigate the roles played by the liposome, peptide and DNA in forming the different complexes. This was completed by systematically probing the circular and linear dichroism spectra of the different components individually, in binary mixtures and finally in the ternary LPD complex to gain an understanding of how the different components of the LPD vector interact with one another.

**Figure 5:**
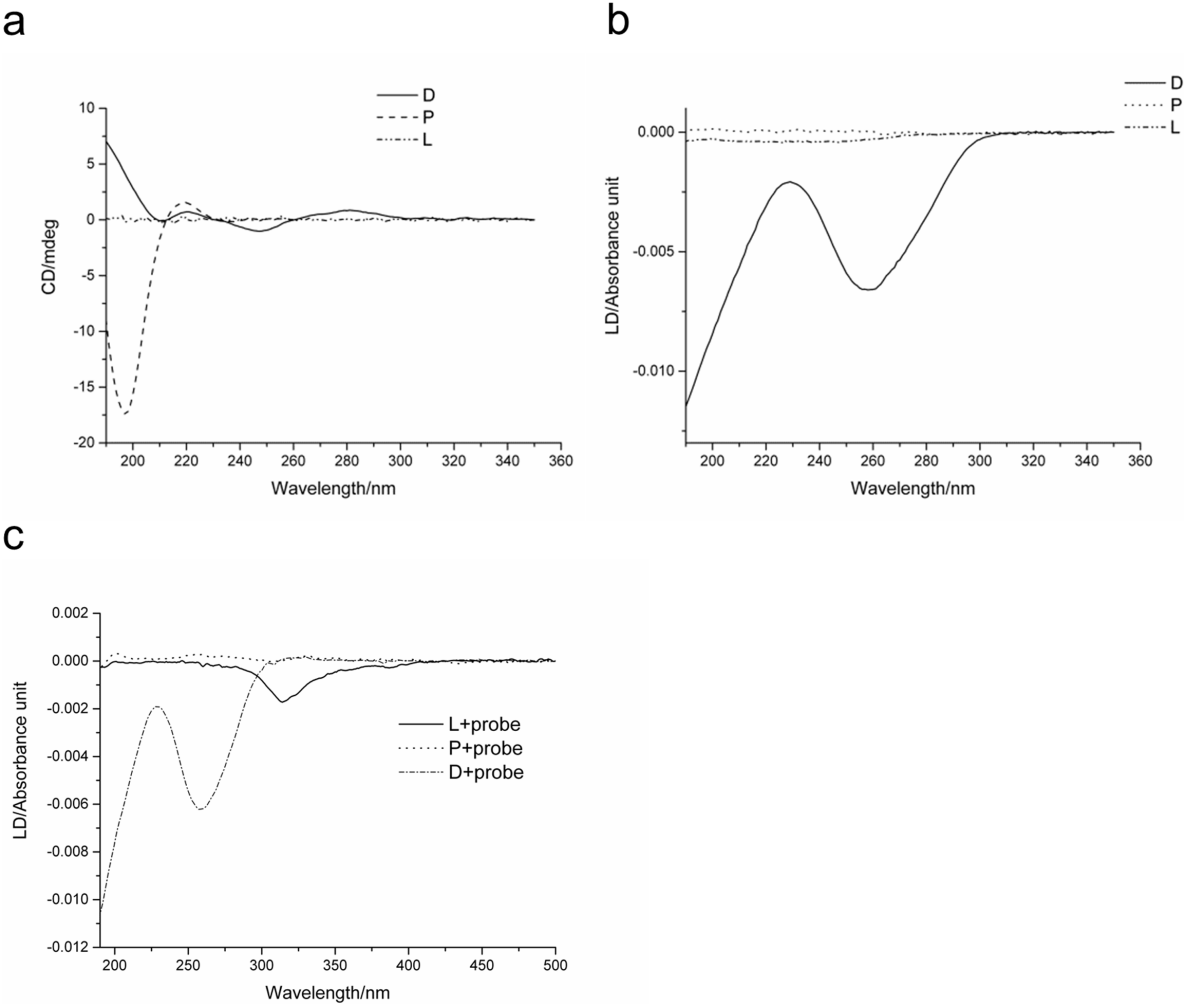
(a) Circular dichroism and (b) linear dichroism spectra of individual components of the LPD complex in water. (c) Linear dichroism spectra of the individual components of the LPD complex following the addition of β-DPH HPC.

Circular (Figure 5a) and linear (Figure 5b) dichroism spectra for plasmid DNA alone were indicative of relaxed B-form DNA which is typical for DNA in aqueous solution^20^. The circular dichroism spectrum of the peptide had the characteristics of an unfolded conformation with a minimum at ~200 nm and a maximum at ~216 nm (Figure 5a)^21^ while, as expected, the linear dichroism spectrum revealed no appreciable signals for the peptide (Figure 5b).

Neither circular dichroism nor linear dichroism signals were obtained for liposome samples due to the absence of chromophores absorbing in the UV region (Figure 5a and b). Linear dichroism spectra for liposomes in the presence of β-DPH HPC, a molecular probe which inserts into lipid bilayers to reveal liposome orientation, had a minimum at ~320 nm (Figure 5c) suggesting that the liposomes were aligned in the shear flow as expected^22^. β-DPH HPC had no effect on the linear dichroism spectrum of peptide or DNA (Figure 5c) suggesting that neither interacted with the probe.

**Figure 6:**
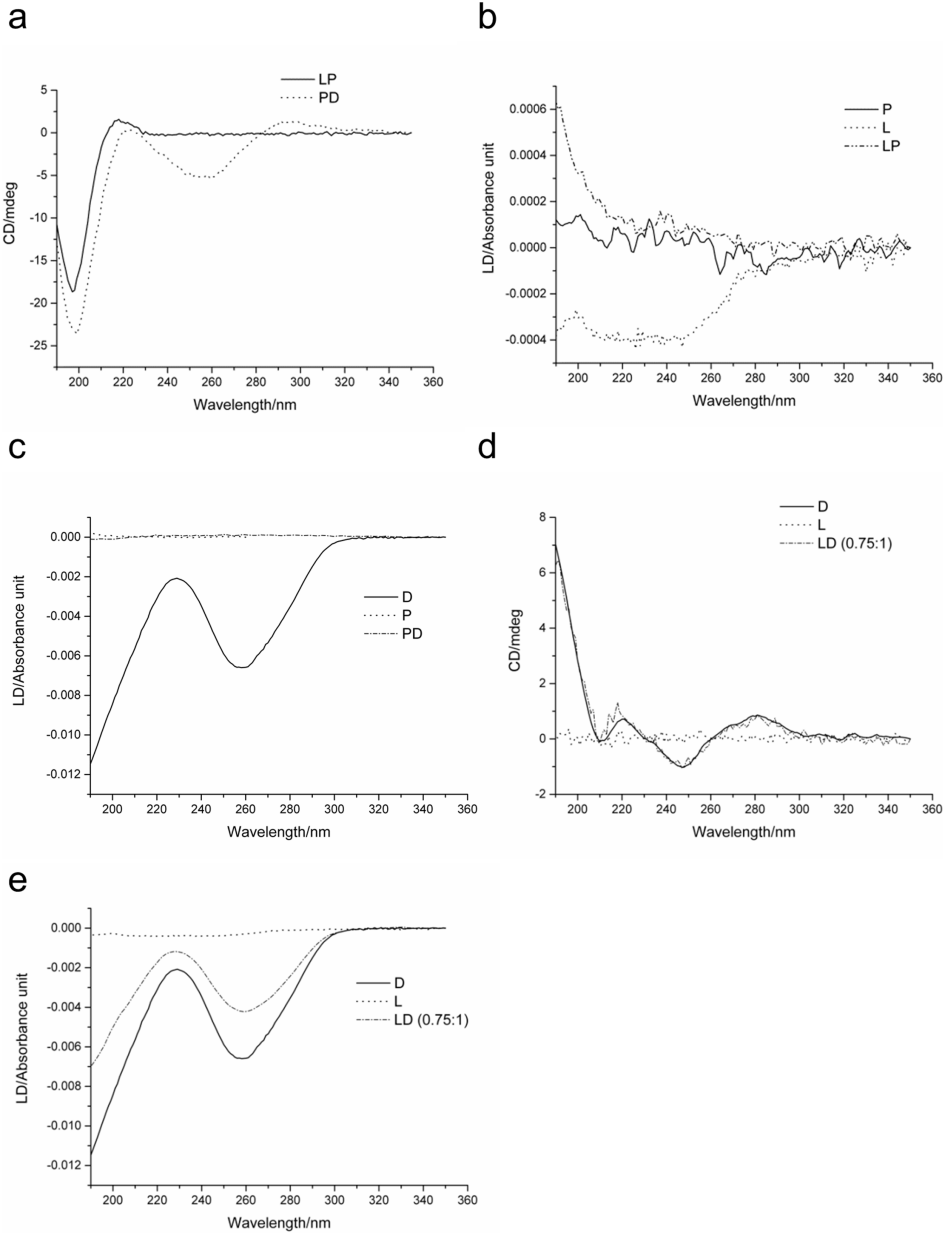
(a) Circular dichroism spectra of liposome-peptide (LP) formulation and PD complexes. (b) Linear dichroism spectra of LP formulation overlaid with linear dichroism spectra of individual liposome (L) and peptide (P). (c) Linear dichroism spectra of PD complexes overlaid with linear dichroism spectra of individual plasmid DNA (D) and peptide (P). (d) Circular dichroism spectra of LD (0.75:1) complexes overlaid with circular dichroism spectra of individual liposome (L) and plasmid DNA (D). (e)Linear dichroism spectra of LD (0.75:1) complexes overlaid with linear dichroism spectra of individual L and D.

There was no evidence for peptide-lipid interactions in binary LP mixtures (liposome: peptide ratio of 1:5.3) from either spectrum (Figure 6a and b). The circular dichroism spectra for PD complexes (Figure 6a) did not reveal notable changes in peptide or DNA structures and appeared to be averages of circular dichroism spectra of the individual components. However, the flattening of the linear dichroism spectrum for DNA (Figure 6c) in PD complexes indicated non-specific interactions, such as electrostatic complexation, rather than co-folding^23^. LD (0.75:1) complexes also showed no apparent changes in DNA structure (Figure 6d and e) and highlighted the relatively poor DNA condensation in LD complexes (Figure 6e).

**Figure 7:**
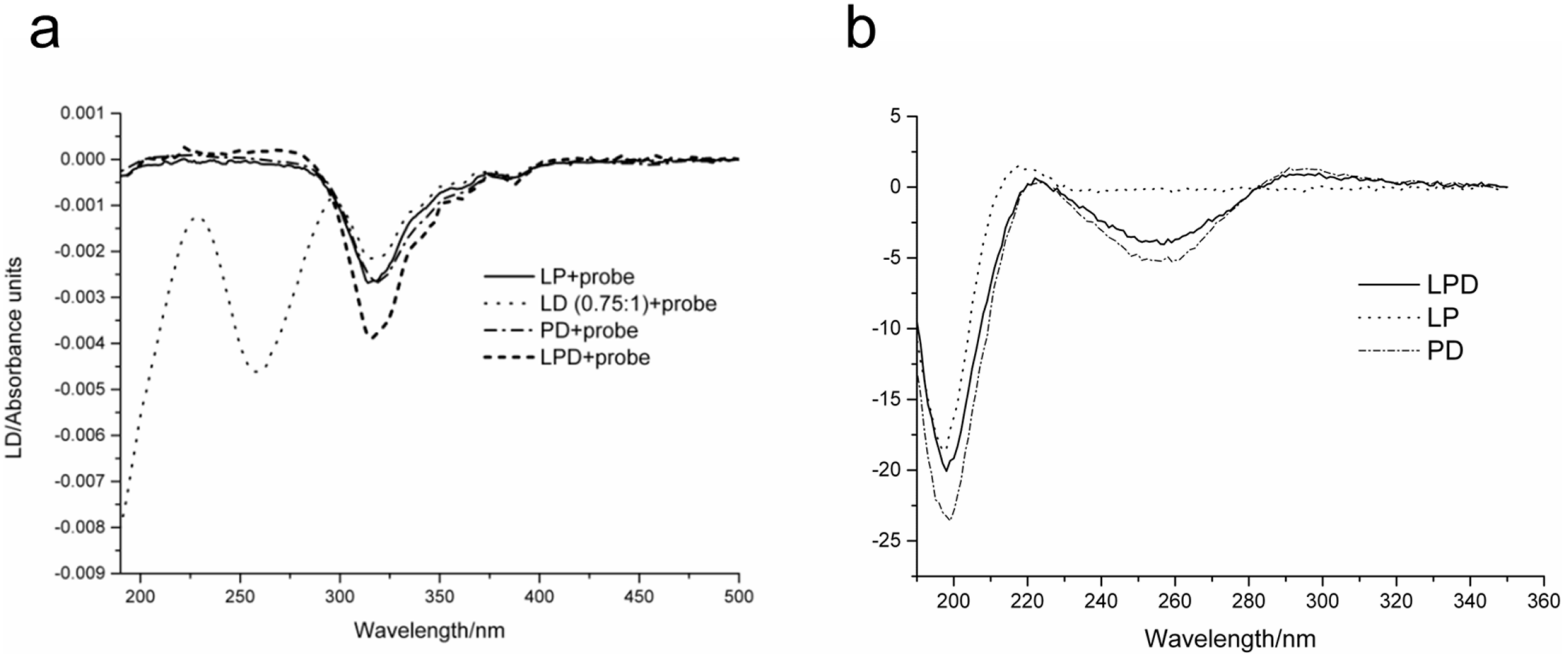
(a) Linear dichroism spectra of LP formulation and LD (0.75:1), PD, and LPD complexes. (b) Circular dichroism spectra LPD and PD complexes and LP formulation.

Addition of the β-DPH HPC probe into different binary mixtures of all three individual components gave rise to the same characteristic band at ~320 nm with increases in intensity for LD(0.75:1) < LP ≤ PD, whereas LPD showed appreciably stronger signals (Figure 7a). The observed increases suggest that the probe integrates more strongly into nanocomplexes with a more pronounced internal order. This is important for two reasons. Firstly, because the probe does not interact with individual peptide and DNA components, but does with liposomes and complexes, it is the supramolecular arrangements of individual components that support probe-complex interactions. Secondly, because the binding of the probe is predominantly driven by the hydrophobic effect inter-component interactions in the complexes set up exposed hydrophobic regions (DNA bases, hydrophobic amino-acid side chains, lipid aliphatic chains) that are sufficiently extensive to support continuous probe binding. The hydrophobic nature of probe-complex interactions is further confirmed by dynamic light scattering (Table 1), which showed no correlations between the sizes and charges of the complexes. In this notation, the linear dichroism data suggests that PD complexes provide stronger hydrophobic interactions than LD. Signals for LPD, which are approximately two-fold stronger than those for LD or PD, are likely to comprise contributions from both types of interactions, LD and PD. Essentially the same circular dichroism spectra for LPD and PD complexes (Fig. 7b) imply that hydrophobic exposure is not a result of folding-mediated assembly and does not necessarily lead to more stable formulations.

## Discussion

Lipopolyplex vectors comprising formulations of liposomes, cationic targeting peptides and DNA (LPD) show significantly higher transfection efficiencies than their liposome-DNA (LD) lipoplex or peptide-DNA (PD) polyplex equivalents, and this has also been observed with other lipopolyplex formulations^6^,^7^,^8^,^9^,^11^. The enhancement of transfection efficiency of LPD formulations is more than an additive effect of LD and PD transfections, suggesting a co-operative effect between the liposome and peptide components in the enhancement of transfection. To better understand the enhanced transfection efficiency properties of LPD vectors we undertook a systematic analysis of the biological and biophysical properties of binary LD and PD complexes and the ternary LPD complex and investigated the roles of the liposome, peptide and DNA components in the formation and function of the LPD vector.

In the transfection process several key steps must take place to allow for efficient gene expression including, i) the formation of stable particles that can protect plasmid DNA from degradation, ii) attachment of vector to cells, iii) uptake of the vector into cells, iv) endosomal escape, v) nuclear entry and vi) release of plasmid DNA from the nanocomplex for transcription.

Our data show that the formation of small discrete cationic particles is a consequence of peptide/DNA interactions in the LPD complexes. We show that cationic peptides more efficiently condense and package DNA than cationic liposomes, a phenomenon seen in previous studies and most likely due to the higher charge density of the peptide molecules^24^. In addition, peptide containing complexes were more stable to heparan sulphate challenge indicating greater protection to the packaged DNA.

Attachment of vector to cells is determined primarily by the surface charge of the vector with cationic complexes being attracted to the anionic cell surface and differences could affect cell binding and vector internalisation^25^. In contrast to the peptide component the amount of liposome used in the LPD complexes was not sufficient to neutralise plasmid DNA highlighting that the formation of cationic particles is also a function of the peptide component.

Cellular uptake follows vector binding and we show that PD and LPD nanocomplexes were internalised to similar degrees whilst, relative to LPD and PD complexes, LD (4:1) complexes showed poor cellular uptake. These differences could be explained by the fact that PD and LPD formulations formed small, spherical and discrete particles in contrast to LD formulations which generally formed large aggregates^26^. Differences in cellular uptake when comparing LPD and LD (4:1) complexes correlated well with differences in luciferase gene expression suggesting that cellular uptake was the primary barrier to LD transfections.

LPD and PD complexes formed nanoparticles with similar biophysical characteristics and displayed good cellular uptake so we hypothesised that the differences in their transfection efficiencies lay in differences in their ability to overcome intracellular barriers to transfection. Endosomal escape and nuclear uptake are downstream of vector uptake and potential barriers hindering efficient transgene expression following PD transfections. Since these studies were performed in dividing cell cultures the nuclear envelope is less of a barrier so we proposed that the greater transfection efficiency of LPD was due to improved endosomal escape mediated by the fusogenic DOPE lipid within the LPD nanocomplex. This hypothesis was supported by the finding that replacement of DOPE in the LPD nanocomplex with the non-fusogenic DOPC lipid eradicated differences in transfection efficiency of LPD and PD complexes.

A further potential limiting factor in transfection efficiency is the dissociation potential of DNA from nanocomplexes within the cell which is necessary to enable access to the transcriptional machinery^27^,^28^,^29^. Our data show that plasmid DNA in PD complexes are more tightly packaged than in LPD complexes while DNA was more easily released from LPD complexes than from PD complexes. Linear and circular dichroism showed a weaker peptide-DNA interaction in LPD complexes suggesting that the cationic liposome interferes with the peptide-DNA interaction in LPD complexes either through steric effects or possibly by competing for interaction with anionic residues on the phosphate backbone of the plasmid DNA. This interference with peptide-DNA interactions in LPD nanocomplexes would lead to reduced water exclusion, charge neutralisation and compaction of the DNA that manifests as slightly larger particles as was observed. The weakening of the peptide-DNA interaction would also make it easier for anionic species within the cells to induce dissociation of the nanocomplex^27^,^28^,^29^ so enabling greater access of DNA to the transcriptional machinery potentially contributing to enhanced transgene expression.

In conclusion, using a lipopolyplex optimised for airway gene delivery we have shown that lipopolyplexes show a synergistic enhancement in the efficiency of transgene expression when compared to their relevant polyplexes and lipoplexes. Cellular uptake of the nanocomplexes was the primary barrier to gene transfer with the lipoplex whilst inefficient intracellular trafficking hampered transgene expression by the polyplex. Having both peptide and liposome components in the lipopolyplex produced a cationic nanocomplex that showed efficient cellular uptake, a function of the peptide component, as well as efficient endosomal escape, a function of the liposome component. This increased functionality afforded by having both the liposome and peptide in lipopolyplexes can help explain the synergy observed in transgene expression when comparing lipoplexes, polyplexes and lipopolyplexes.

## Methods

### Materials

Plasmids pCI-Luc and pEFGP-N1 were prepared as previously described^12^. Plasmid pCI-Luc comprises the luciferase reporter gene sub-cloned into the eukaryotic expression vector pCI (Promega, Southampton, UK) with transcription driven by the cytomegalovirus immediate/early promoter-enhancer^13^. The plasmid pEGFP-N1 was purchased from Clontech (Saint-Germain-en-Laye, France). Peptide K_16_GACSERSMNFCG^19^ was synthesised by Zinsser Analytic (Maidenhead, UK), and dissolved in endotoxin-free water (Sigma-Aldrich, Dorset, UK) to 10 mg/mL. Liposomes consisted of 1-Propanaminium, *N,N,N*-trimethyl-2,3-bis(11*Z*-hexadecenyloxy)-iodide (DHDTMA iodide; Avanti Polar Lipids; Alabama, USA) with either dioleoylphosphatidylethanolamine (DOPE; Avanti Polar Lipids; Alabama, USA) or dioleoylphosphatidylcholine (DOPC; Avanti Polar Lipids; Alabama, USA) formulated at a 1:1 weight ratio, dissolved in sterile water to give a 2 mg/mL liposome suspension.

### Cell culture

16HBE14o- cells (human bronchial epithelia origin; kind gift from Dieter Gruenert, San Francisco^30^) were cultured in complete media consisting of Eagle's Minimal Essential Medium (MEM; Sigma-Aldrich, Dorset, UK) supplemented with 10% (v/v) foetal bovine serum, 2 mmol/l L-glutamine. HEK-293T and HT-1080 cells were cultured in Dulbecco's Modified Eagle Medium (DMEM; Life Technologies, UK) supplemented with 10% (v/v) foetal bovine serum. Cells were incubated in humidified 5% CO_2_ at 37°C.

### In vitro transfections

Cells were seeded in 96-well plates at a density of 20,000 cells per well and incubated in humidified 5% CO_2_ at 37°C for 24 hours. For luciferase assay experiments cells were seeded in black plates with clear bottoms (Fisher Scientific UK, Leicestershire, UK). All complexes were prepared in OptiMEM (Invitrogen, Paisley, UK). LPD complexes were prepared at a 0.75:4:1 weight ratio of liposome: peptide: plasmid DNA^12^; PD complexes were formulated at a 4:1 peptide: plasmid DNA weight ratio and LD complexes were prepared either at a 0.75:1 (LD (0.75:1)) or 4:1 (LD (4:1)) weight ratio of liposome: plasmid DNA. Complexes were formulated to a plasmid DNA concentration of 10 μg/mL and incubated at room temperature for 30 minutes to allow for complex self-assembly. LPD formulations were prepared by first mixing the cationic liposome and peptide solutions and then adding the plasmid DNA solution. Cells were transfected in replicates with 25 μL of the formulated complex solution (250 ng plasmid DNA) added to 175 μL of complete media per well. Luciferase and protein assays were performed 24 hours following transfection.

### Luciferase assay

Cells were washed with 1× PBS 24 hours following transfection and lysed with 20 μL Reporter Lysis Buffer (Promega, Southampton, UK) for 20 minutes at 4°C then −80°C for at least 30 minutes followed by thawing at room temperature. Luciferase activity was assessed using the Luciferase Assay System (Promega, Southampton, UK) on a FLUOstar Optima plate reader (BMG Labtech, Aylesbury, UK). The results were standardised for protein content using the Bradford protein assay (Bio-Rad, Hertfordshire, UK) and expressed as relative luminescence units (RLU/mg of protein).

### Cell uptake of Cy5 labelled plasmid

pCI-Luc plasmid was labelled with Cy5 using the Universal Linkage System Nucleic Acid Labelling Kit (Kreatech Diagnostics, Amsterdam, The Netherlands). Labelled plasmid was mixed 1:4 with unlabelled pCI-Luc plasmid and complexed into LD (0.75:1), LD (4:1), PD (4:1) and LPD and used for transfection of 16HBE14o^-^ cells and the cells were then incubated with the complexes in humidified 5% CO_2_ at 37°C for 6 hours and then processed for flow cytometry and laser scanning confocal microscopy.

### Flow cytometry

Adherent cells in 96 well plates were detached using 50 μL Trypsin-EDTA (Sigma-Aldrich, Dorset, UK) and re-suspended with 150 μL DPBS (Sigma-Aldrich, Dorset, UK). Cells were then acquired with a BD FACSArray flow cytometer (BD Biosciences Oxford, UK) and analysis was performed with FlowJo software v. 8.8.3 (Tree Star Inc., Olten, Switzerland).

### Laser scanning confocal microscopy

Transfected cells were fixed with 4% paraformaldehyde, and the nucleus and F-actin were stained with 4',6-diamidino-2-phenylindole (DAPI) and Alexa Fluor 488 Phalloidin (Life Technologies, Paisley, UK) respectively. Images were obtained using a Zeiss LSM 710 Confocal (Carl Zeiss Ltd., Cambridge, UK).

### Electron microscopy

LD (0.75:1), LD (4:1), PD, and LPD complexes were prepared as above for transfections except in water rather than OptiMEM. A 5 μL aliquot was applied onto a 300-mesh copper grid coated with a Formvar/carbon support film (Agar Scientific, Essex, UK) then, after a few seconds, dried by blotting with filter paper. The sample was then negatively stained with 1% uranyl acetate for a few seconds, before blotting with filter paper and air dried. Imaging was carried out with a Philips CM120 BioTwin Transmission Electron Microscope and operated at an accelerating voltage of 120 kV.

### Hydrodynamic size and zeta potential measurements

Aqueous preparations of LD (0.75:1), LD (4:1), PD, and LPD complexes prepared as for electron microscopy above were diluted to a final volume of 1 mL in distilled water at a concentration of 5 μg/mL plasmid DNA and then analysed for hydrodynamic size and surface charge (ζ potential) using a Malvern Nano ZS (Malvern, Worcestershire, UK). Aqueous preparations of liposome alone and a liposome-peptide mixture (LP; liposome: peptide ratio of 1:5.3) were also diluted in a final volume of 1 mL in distilled water at a concentration of 5 μg/mL of liposome before being analysed as above. The data was collected and processed by the manufacturer's software, DTS version 5.03.

### Gel retardation assay

LD (0.75:1), LD (4:1), PD, and LPD complexes were prepared in water, then some formulations were treated with 10 U/mL heparan sulphate (Sigma-Aldrich, Dorset, UK) for 1 hour at room temperature. Samples containing 250 ng of plasmid DNA complexed into particles (or plasmid DNA alone as a control) were loaded onto a 1% agarose gel, made in Tris-acetate-EDTA (TAE) buffer and stained with 1 μg/mL ethidium bromide, and electrophoresed at a voltage of 100 V for 1 hour with TAE as the running buffer.

### PicoGreen fluorescence quenching

250 ng of plasmid DNA was mixed with PicoGreen reagent (1:150; Invitrogen, Paisley UK) in TE buffer and incubated for 5 minutes at room temperature. The stained plasmid DNA was then formulated into LD (0.75:1), LD (4:1), PD, and LPD complexes as above but using TE buffer. Varying concentrations of heparan sulphate (0–10 U/mL; Sigma-Aldrich, Dorset, UK) diluted in TE buffer were then mixed with the complexes and incubated for a further hour at room temperature then fluorescence was analysed at excitation and emission wavelengths of 485 nm and 520 nm, respectively using the FLUOstar Optima plate reader (BMG Labtech, Aylesbury, UK). Values were collected as relative fluorescence units (RFU) and these were then normalised to naked plasmid DNA that was stained with PicoGreen and treated with the varying concentrations of heparan sulphate.

### Circular dichroism

Circular dichroism measurements were performed with aqueous preparations of LD (0.75:1), LD (4:1), PD, and LPD complexes, as well as liposome, peptide and plasmid DNA components at concentrations consistent with those for the LPD complex. The individual components were used as controls to determine their native structures. All spectra were obtained in a JASCO J-810 spectropolarimeter using a 0.1 cm rectangular quartz cuvette cell, at a scan rate of 50 nm/min, data pitch of 1 nm, response time of 1 s and bandwidth of 1 nm.

### Linear dichroism

LD (0.75:1), LD (4:1), PD, and LPD complexes were formulated as above for circular dichroism. Liposome, peptide and plasmid DNA were used as controls to probe the orientation of the individual components under laminar flow. A 1 mg/mL solution of β-DPH-HPC, (2-(3-(diphenylhexatrienyl)propanpyl)-1-hexadecanoyl-sn-glycero-3-phosphocholine; Invitrogen, Paisley, UK), in ethanol was, where stated, added to different formulations at a final concentration of 12.5 μg/mL. The probe incorporates into the liposome bilayer and orients with the liposome thus enabling the determination of the relative orientation of the liposome from the linear dichroism signal of β-DPH-HPC^22^.

Solution-phase flow linear dichroism spectra were obtained on the same Jasco-810 spectropolarimeter as for circular dichroism, which was adapted by substituting the photo elastic modulator (PEM) 1/2 wave plate instead of a 1/4 wave plate^22^,^31^. Linear dichroism spectra were acquired in a quartz Couette flow cell of ~0.5 mm annular gap (Kromatec Ltd, UK). Molecular alignment was achieved through the constant flow of the sample solution between two coaxial cylinders – a stationary quartz rod and a rotating cylindrical capillary. The laminar flow for the efficient alignment was obtained by maintaining the rotation speed at ~3000 rpm and a non-rotating baseline spectrum was subtracted^22^,^32^,^33^. The linear dichroism spectra were the average of four accumulations in continuous mode using a scan rate of 50 nm/min, data pitch of 1 nm, response time of 1 s and bandwidth of 2 nm.

### Statistical analysis

Data are mean values ± standard error of the mean (SEM). A one-way ANOVA with Bonferroni post-hoc test was used to assess statistical significance. Significance levels were set at *p* < 0.05. * = *p <* 0.05; ** = *p <* 0.01; *** = *p <* 0.001; NS = not significant.

## Author Contributions

M.M.M., J.R. and A.D.T. designed and performed experiments, analysed data and prepared the manuscript. D.M. performed experiments. M.G.R and S.L.H. designed the study and supervised the project. All authors discussed the results and commented on the manuscript.

## Supplementary Material

Supplementary Information Figure S1-S4

## Figures and Tables

**Table 1:** Hydrodynamic size and zeta potential of complexes

Nanocomplex	Size (nm)	Zeta potential (mV)
LPD	58.0 ± 0.2	+67.2 ± 2.1
LD (0.75:1)	116.1 ± 1.1	−20.8 ± 0.2
LD (4:1)	280.3 ± 1.0	+32.5 ± 0.5
PD	44.6 ± 0.1	+31.0 ± 0.6
Liposome	82.1 ± 1.1	+60.1 ± 0.9
LP	86.6 ± 0.5	+74.9 ± 1.7

## References

[b1] Lamarre B. & Ryadnov M. G. Self-assembling viral mimetics: one long journey with short steps. Macromol. Biosci. 11, 503–513 (2011). 21165940 10.1002/mabi.201000330

[b2] Felgner P. L. *et al.* Lipofection: a highly efficient, lipid-mediated DNA-transfection procedure. Proc. Natl. Acad. Sci. U. S. A. 84, 7413–7417 (1987). 2823261 10.1073/pnas.84.21.7413 PMC299306

[b3] Wu G. Y. & Wu C. H. Receptor-Mediated Invitro Gene Transformation by a Soluble DNA Carrier System. J. Biol. Chem. 262, 4429–4432 (1987). 3558345

[b4] de Ilarduya C. T., Sun Y. & Duezguenes N. Gene delivery by lipoplexes and polyplexes. Eur. J. Pharm. Sci. 40, 159–170 (2010). 20359532 10.1016/j.ejps.2010.03.019

[b5] Edinger D. & Wagner E. Bioresponsive polymers for the delivery of therapeutic nucleic acids. Wiley Interdiscip. Rev. Nanomed. Nanobiotechnol. 3, 33–46 (2011). 20533517 10.1002/wnan.97

[b6] Wang B. *et al.* Chitosan enhanced gene delivery of cationic liposome via non-covalent conjugation. Biotechnol. Lett. 34, 19–28 (2012). 22009568 10.1007/s10529-011-0748-8

[b7] Kurosaki T. *et al.* Pulmonary gene delivery of hybrid vector, lipopolyplex containing N-lauroylsarcosine, via the systemic route. J. Control. Release 136, 213–219 (2009). 19233236 10.1016/j.jconrel.2009.02.005

[b8] Garcia L., Bunuales M., Duzgunes N. & de Ilarduya C. T. Serum-resistant lipopolyplexes for gene delivery to liver tumour cells. Eur. J. Pharm. Biopharm. 67, 58–66 (2007). 17321729 10.1016/j.ejpb.2007.01.005

[b9] Collins E., Birchall J. C., Williams J. L. & Gumbleton M. Nuclear localisation and pDNA condensation in non-viral gene delivery. J. Gene Med. 9, 265–274 (2007). 17397103 10.1002/jgm.1015

[b10] Whitmore M., Li S. & Huang L. LPD lipopolyplex initiates a potent cytokine response and inhibits tumor growth. Gene Ther. 6, 1867–1875 (1999). 10602382 10.1038/sj.gt.3301026

[b11] Hart S. L. *et al.* Lipid-mediated enhancement of transfection by a nonviral integrin-targeting vector. Hum. Gene. Ther. 9, 575–585 (1998). 9525318 10.1089/hum.1998.9.4-575

[b12] Tagalakis A. D. *et al.* A receptor-targeted nanocomplex vector system optimized for respiratory gene transfer. Mol. Ther. 16, 907–915 (2008). 18388925 10.1038/mt.2008.38

[b13] Jenkins R. G. *et al.* Formation of LID vector complexes in water alters physicochemical properties and enhances pulmonary gene expression in vivo. Gene Ther. 10, 1026–1034 (2003). 12776160 10.1038/sj.gt.3301963

[b14] Irvine S. A. *et al.* Receptor-targeted nanocomplexes optimized for gene transfer to primary vascular cells and explant cultures of rabbit aorta. Mol. Ther. 16, 508–515 (2008). 18180778 10.1038/sj.mt.6300381

[b15] Meng Q. H., Jamal W., Hart S. L. & McEwan J. R. Application to vascular adventitia of a nonviral vector for TIMP-1 gene therapy to prevent intimal hyperplasia. Hum. Gene. Ther. 17, 717-727 (2006). 16839271 10.1089/hum.2006.17.717

[b16] Meng Q. H. *et al.* Inhibition of neointimal hyperplasia in a rabbit vein graft model following non-viral transfection with human iNOS cDNA. Gene Ther. 20, 979–986 (2013). 23636244 10.1038/gt.2013.20 PMC3795475

[b17] Grosse S. M. *et al.* Tumor-specific gene transfer with receptor-mediated nanocomplexes modified by polyethylene glycol shielding and endosomally cleavable lipid and peptide linkers. FASEB J. 24, 2301–2313 (2010). 20203088 10.1096/fj.09-144220

[b18] Tagalakis A. D. *et al.* Integrin-targeted nanocomplexes for tumour specific delivery and therapy by systemic administration. Biomaterials 32, 1370–1376 (2011). 21074847 10.1016/j.biomaterials.2010.10.037

[b19] Writer M. J. *et al.* Targeted gene delivery to human airway epithelial cells with synthetic vectors incorporating novel targeting peptides selected by phage display. J. Drug Target. 12, 185–193 (2004). 15506167 10.1080/10611860410001724459

[b20] Ido S. *et al.* Beyond the helix pitch: direct visualization of native DNA in aqueous solution. ACS Nano 7, 1817–1822 (2013). 23350676 10.1021/nn400071n

[b21] Norden B. & Kurucsev T. Analysing DNA complexes by circular and linear dichroism. J. Mol. Recognit. 7, 141–155 (1994). 7826674 10.1002/jmr.300070211

[b22] Hicks M. R. *et al.* Synchrotron radiation linear dichroism spectroscopy of the antibiotic peptide gramicidin in lipid membranes. Analyst 134, 1623–1628 (2009). 20448930 10.1039/b902523e

[b23] Johnson W. C. in Circular dichroism and the conformational analysis of biomolecules (ed Fasman, G. D.) 433–468 (Plenum Press, 1996).

[b24] Elouahabi A. & Ruysschaert J. M. Formation and intracellular trafficking of lipoplexes and polyplexes. Mol. Ther. 11, 336–347 (2005). 15727930 10.1016/j.ymthe.2004.12.006

[b25] Hui S. W. *et al.* The role of helper lipids in cationic liposome-mediated gene transfer. Biophys. J. 71, 590–599 (1996). 8842198 10.1016/S0006-3495(96)79309-8 PMC1233516

[b26] Rejman J., Oberle V., Zuhorn I. S. & Hoekstra D. Size-dependent internalization of particles via the pathways of clathrin- and caveolae-mediated endocytosis. Biochem. J. 377, 159–169 (2004). 14505488 10.1042/BJ20031253 PMC1223843

[b27] Mannisto M. *et al.* Potyptex-mediated gene transfer and cell cycle: effect of carrier on cellular uptake and intracellular kinetics, and significance of glycosaminoglycans. J. Gene Med. 9, 479–487 (2007). 17410614 10.1002/jgm.1035

[b28] Tagalakis A. D., He L., Saraiva L., Gustafsson K. T. & Hart S. L. Receptor-targeted liposome-peptide nanocomplexes for siRNA delivery. Biomaterials 32, 6302–6315 (2011). 21624650 10.1016/j.biomaterials.2011.05.022

[b29] Tagalakis A. D., Saraiva L., McCarthy D., Gustafsson K. T. & Hart S. L. Comparison of Nanocomplexes with Branched and Linear Peptides for SiRNA Delivery. Biomacromolecules 14, 761–770 (2013). 23339543 10.1021/bm301842j

[b30] Cozens A. L. *et al.* CFTR expression and chloride secretion in polarized immortal human bronchial epithelial cells. Am. J. Respir. Cell Mol. Biol. 10, 38–47 (1994). 7507342 10.1165/ajrcmb.10.1.7507342

[b31] Rodger A. *et al.* Looking at long molecules in solution: what happens when they are subjected to Couette flow? Phys. Chem. Chem. Phys. 8, 3161–3171 (2006). 16902709 10.1039/b604810m

[b32] Marrington R. *et al.* Validation of new microvolume Couette flow linear dichroism cells. Analyst 130, 1608–1616 (2005). 16284659 10.1039/b506149k

[b33] Dafforn T. R., Rajendra J., Halsall D. J., Serpell L. C. & Rodger A. Protein fiber linear dichroism for structure determination and kinetics in a low-volume, low-wavelength couette flow cell. Biophys. J. 86, 404–410 (2004). 14695282 10.1016/S0006-3495(04)74116-8 PMC1303805

