# Supplementary material for: Role of liposome and peptide in the synergistic enhancement of transfection with a lipopolyplex vector
Source: Sci Rep. 2015 Mar 19;5:9292. doi: 10.1038/srep09292 (PMC4365389; doi:10.1038/srep09292)
Supplement: Supplementary Information — Figure S1-S4 [file srep09292-s1.pdf]

Role of liposome and peptide in the synergistic enhancement of transfection with a lipopolyplex vector

Munye MM[1] *; Ravi J[2]; Tagalakis AD[1]; McCarthy D[3]; Ryadnov MG[2]; Hart SL[1]

[1] UCL Institute of Child Health, 30 Guilford Street, London, WC1N 1EH, United Kingdom

[2]National Physical Laboratory, Teddington, Middlesex, TW11 0LW, United Kingdom

[3] UCL School of Pharmacy, 29-39 Brunswick Square, London, WC1N 1AX, United Kingdom

*Corresponding author: m.munye@ucl.ac.uk

UCL Institute of Child Health, 30 Guilford Street, London, WC1N 1EH, United Kingdom

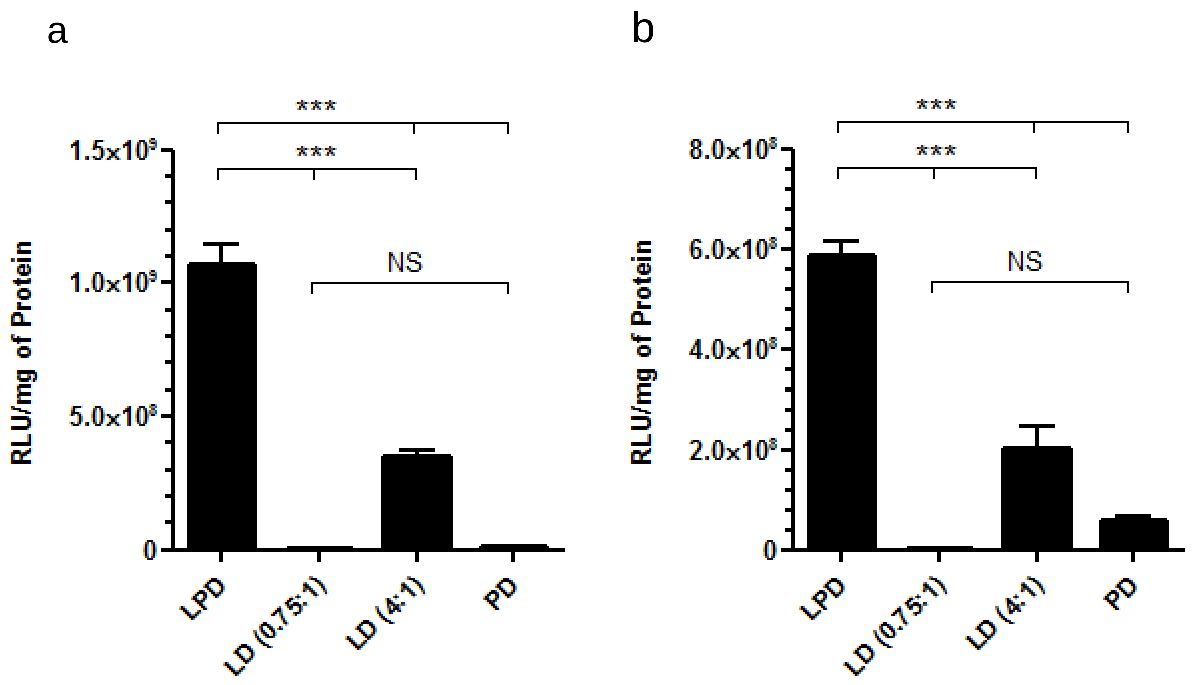

Figure S1. Luciferase activity, measured in relative light units (RLU) per mg of protein following transfection of (a) HEK-293T and (b) HT-1080 cells with pCI-Luc complexed into LPD, LD (0.75:1), LD (4:1) and PD nanocomplexes.

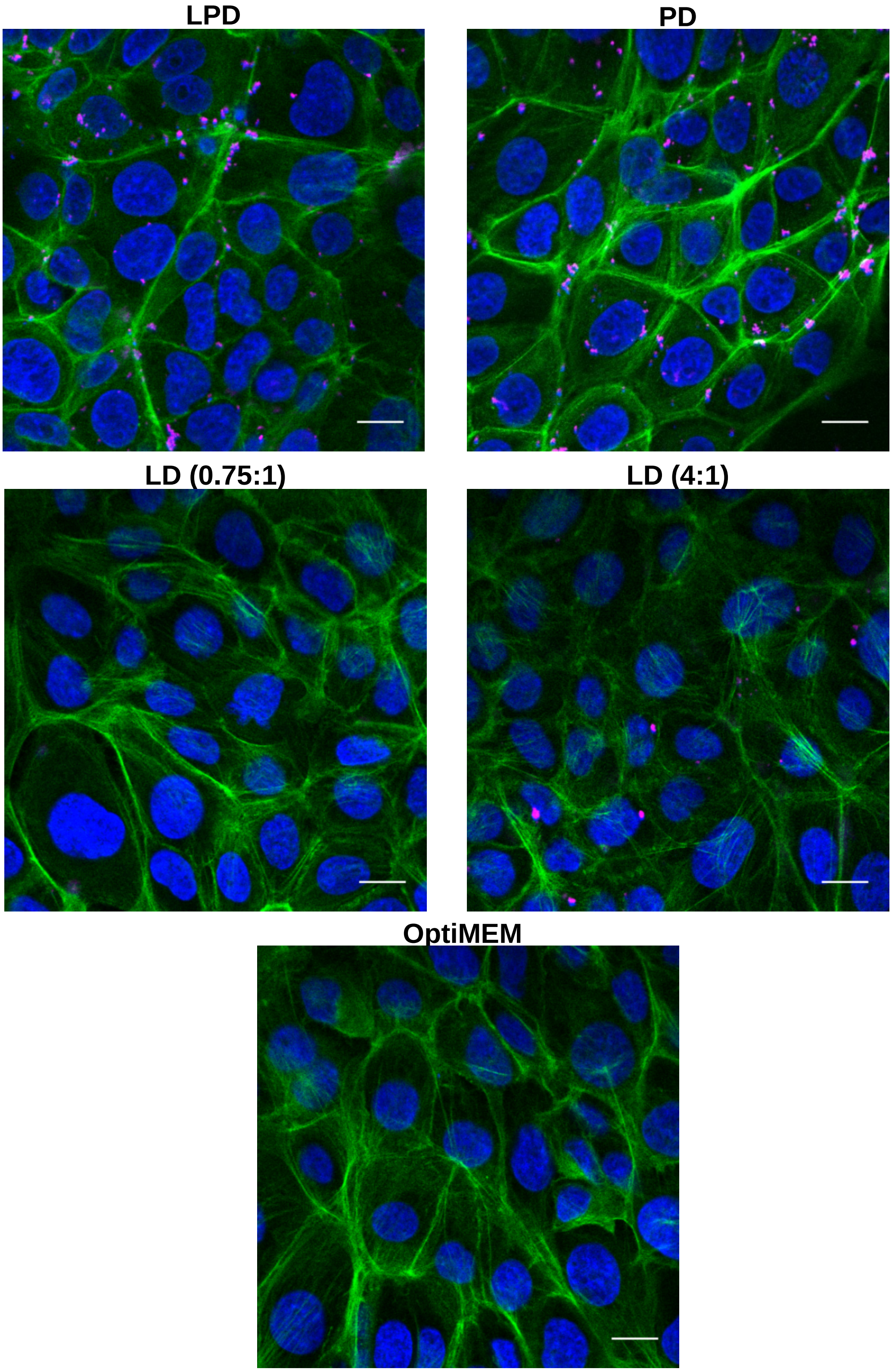

Figure S2. Laser scanning confocal microscopy images of $16HBE14o^-$ cells transfected with Cy5 labelled pCI-Luc (magenta) complexed into LPD, PD, LD (0.75:1) and LD (4:1) nanocomplexes. Control cells were left untransfected in OptiMEM. Nucleus of the cells was stained with DAPI (blue) and F-actin (green) staining was used to show cell boundaries. Scale bars are 15µm.

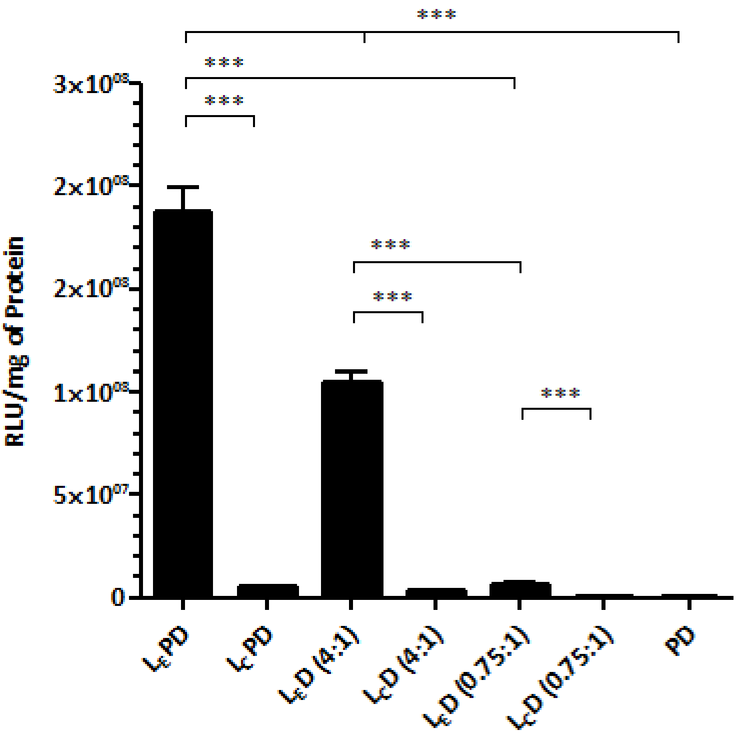

Figure S3. Luciferase activity, measured in relative light units (RLU) per mg of protein following transfection of 16HBE14o^{-} cells with pCI-Luc complexed into LPD, LD (0.75:1), LD (4:1) and PD nanocomplexes formulated with DHDTMA:DOPE (L_E) or DHDTMA:DOPC (L_C) liposomes.

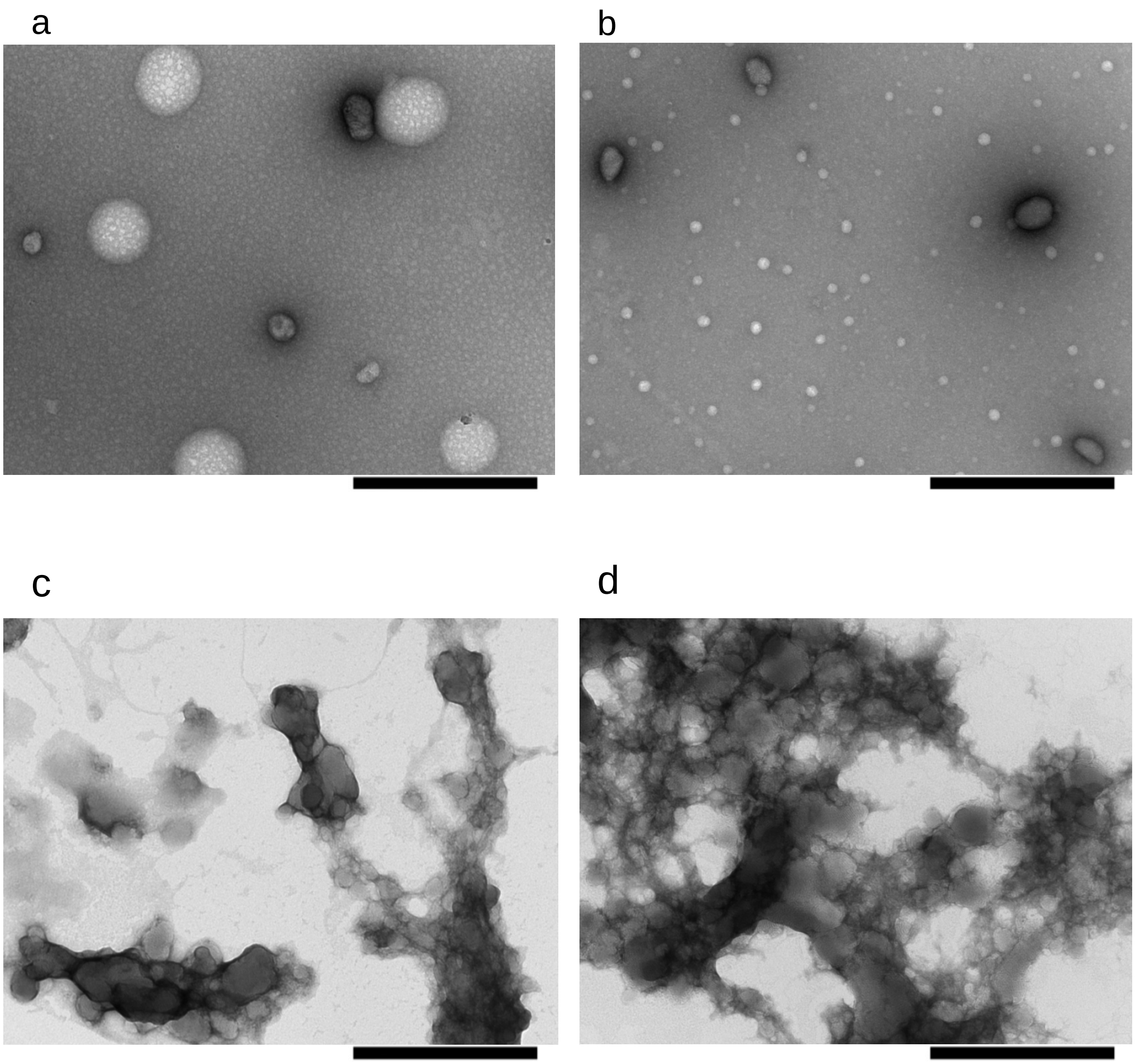

Figure S4. Transmission electron micrographs of (a) LPD and (b) PD (c) LD (0.75:1) and (d) LD (4:1) complexes. Scale bars are 500nm.
